# Effect of Different Fertilizations on the Plant-Available Nitrogen in Soil Profile (0–100 cm): A Study on Chinese Cabbage

**DOI:** 10.3389/fpls.2022.863760

**Published:** 2022-04-11

**Authors:** Rasheed Ahmed, Lili Mao, Yuzhong Li, Junjun Ding, Wei Lin, Shakeel Ahmed, Asad Abbas, Waseem Ahmed

**Affiliations:** ^1^Institute of Environment and Sustainable Development in Agriculture, Chinese Academy of Agricultural Sciences, Beijing, China; ^2^School of Environment, Tsinghua University, Beijing, China; ^3^School of Horticulture, Anhui Agricultural University, Hefei, China; ^4^Institute of Horticultural Sciences, University of Agriculture, Faisalabad, Pakistan; ^5^Department of Horticulture, University of Haripur, Haripur, Pakistan

**Keywords:** nitrate, ammonia, pH, EC, Chinese cabbage, PAN

## Abstract

The aim of this study is to analyze the variations in the plant-available nitrogen (PAN) concentrations in the soil profile. Different fertilizers were applied for Chinese cabbage plantation (CCP) in the experimental fields of the Shunyi region. The treatments used for the comparative analysis are (i) no fertilizer and plantation (NVP), (ii) no fertilizer with CCP (CTP), (iii) fertilization as urea (URP), and (iv) potassium nitrate (KNP) and chicken manure (CMP) with CCP. It was concluded that the yield was significantly high in URP, CMP, and KNP as compared to CTP. In URP, maximum PAN in soil layers 0–60 cm was recorded during crop production and in 60–100 cm after harvesting as compared to other treatments. Significant variations in soil pH and electrical conductivity (EC) for the soil profile (0–100 cm) from the initial values with respect to time and treatments were observed. CMP showed maximum ammonium in the upper layers of 0–60 cm throughout the season, whereas minimum PAN was observed in NVP but increased in lower layers of 60–100 cm. In general, all fertilizers raised the PAN below the soil 60–100 cm which indicates their potential for nitrate leaching (NL).

## Introduction

Nitrogen (N) is the essential macro-nutrient for plants and is thereby added to agricultural soil to enhance crop production. Soil contains nitrogen in the form of organic and inorganic compounds which are usually in minute amounts with respect to crop demand, thus the application of N fertilizers is considered as a key factor to increase crop yield throughout the world. Globally, N is added in larger quantities, so more N losses transpire to the environment in the form of nitrate leaching (NL) and nitrous oxide emission (Cameron et al., 2013; Yousaf et al., 2016). Nitrate and ammonium ions are the two major forms of N that are consumed by the plants and the preference of one form over another depends upon plant species and environmental factors. These two inorganic pools describe the major portion of plant-available nitrogen (PAN) in soil for crop production and contribute to nitrogen losses (Britto and Kronzucker, 2013; Shah et al., 2017).

Every soil contains nitrate and ammonium concentrations that are the product of soil mineralization of organic matter and applied N fertilizers (Zhang et al., 2013). The mineralization rate in agricultural soil is directly linked with soil pH, temperature, and soil electrical conductivity (EC) (Curtin et al., 1998; Yan et al., 2015; Liu et al., 2016; Gubry-Rangin et al., 2017). Soil nitrate and ammonium are present mostly in lower amounts, hence they are supplemented as N fertilizer and their residence time in the soil root zone mainly depends on the soil texture (Bimüller et al., 2014). Furthermore, N addition more than plant demands may build a peak concentration layer of inorganic N in the soil profile and depends on the fertilizer application and move down with time along with water (Wang et al., 2014; Dai et al., 2015; Van Meter et al., 2016). The gradual leaching of residual nitrate into the layers below the root zone is a major N loss in cropping systems (Chen et al., 2014; Li et al., 2016). Total organic N and inorganic N present in the different horizons of soil from the topsoil to bedrock is a function of depth from the upper surface, time, drainage quantity, and quality passing through the soil profile (Johnsson et al., 1987; Negm et al., 2014). After N fertilization, ammonium ions in upper soil layers increase up to a threshold and then start to reduce with an associated increase in nitrate concentration (Kraft et al., 2014). According to Sullivan et al. (2014), the amount of biologically fixed atmospheric nitrogen added to soil is smaller as compared to the amount of fertilizer added for a specific period of time. Therefore, fertilizer addition is the real cause of NL in many regions of the world.

Nitrate is highly soluble in water and does not bind well with the soil particles because of its negative charge, similar to clay particles. Thus, water movement carries nitrate from the soil medium to surface water (runoff) or groundwater *via* NL (Zhou and Butterbach-Bahl, 2014). Therefore, nitrate is the most important ion related to groundwater pollution due to its high solubility in water and potential for NL (Basso et al., 2013). Higher N application favors the higher mineralization with an ultimate abundance of nitrate ions in the soil causes higher nitrate accumulation in the different parts of the plant body, particularly in vegetables (Colla et al., 2018). Subsequently, this connection may generate peroxynitrite (ONOO^–^) through nitrate reductase in portions of the plant, which is highly toxic to human beings and animals (Islam et al., 2015; Schwertz et al., 2016).

Riley and Decker (2000) found significant variations in the soil nitrate and ammonium levels in the soil profile from 0 to 60 cm under the different types of fertilizers during the wheat cropping season. In addition, the most commonly used fertilizer rate of 250 kg N/ha with irrigation had the potential to raise the nitrate level in soil layers and subsequently cause NL to the deeper layers in the soil profile. Moreover, they concluded that the most commonly used fertilization practices (irrigation and 250 kg N/ha) had the potential to increase the N accumulation in soil layers as well as NL. Zhongjiang et al. (2014) found a significant decrease in ammonium levels from the top 10–15 cm soil with an increase in the nitrate level under different fertilization. Similarly, Yang et al. (2013) studied dry land soil in Shanxi, China and found that the peak nitrate concentration layer is developed at 80 cm in soil profile after longtime straw application. In fact, PAN accumulation in the soil profile below the root zone is the main cause of groundwater nitrate contamination in the Chinese semi-humid croplands because heavy rainfall in the monsoon season or irrigation water moves mineral N deeper in soil layers (Yang et al., 2015; Zhou et al., 2016). A recent study on higher nitrate concentration in the filtered groundwater of Shaanxi and Shandong provinces showed the adverse effects on the resident population (Zhai et al., 2017).

Increased food demand increased chemical fertilization throughout the world, especially in the large populated Asian countries, such as China and India (Liu et al., 2014). According to the results of the Rapid Diagnostic Appraisal survey, vegetables in the Shunyi District were heavily fertilized approximately 2,250 kg N/ha (150 kg N/μ) of organic manure and sometimes combined use with potassium fertilizers (Quan et al., 2015). Poultry industries have been increased multi-fold times since 1980, especially by small farmers as the family-run farming system in China (Fan et al., 2010). These farmers are incorporating the poultry manures abundantly without being concerned about the crop requirements, resulting in the accumulation of inorganic N in the soil profile and ultimately causing to increase NL (Lu et al., 2012; Zheng et al., 2017). Different types of seedbeds are prepared in the crop rotation system, such as a row cropping to cultivate the vegetable and the hill drop or broadcasting for cereal crops. These cultivation practices may leave few places fallow in the agricultural fields. In a similar way, fields are left fallow without crop cultivation in the crop rotation to maintain soil fertility or for any other purpose. These fallow soils in agricultural land have higher N mineralization and nitrate in the upper soil layers. Eventually, this increase in the nitrate level contributes to building a peak concentration deeper in sub-soil or causes to contaminate the groundwater. During seedbed preparation, a bare land section may construct when cereal cropping fields are prepared to grow row cropping vegetables. Similarly, during crop rotation seasons without plantation and fertilization (fallow period) are adjusted to maintain fertility and for any other purpose. According to Chen et al. (2016), bare land may also contribute to significant NL in the agricultural soil in China because of the previous fertilization and rainfall effects (Zheng et al., 2017).

This study was planned to analyze the variations in the PAN with 25–27%, pH, and EC in the soil profile under different fertilization during and after the cropping season of Chinese cabbage (*Brassica rapa* subsp. *pekinensis)* in the experimental fields. The variations in soil parameters were observed with respect to time (cropping season) and layers (0–100 cm) under fertilizer treatments including chicken manure, potassium nitrate and urea, CMP, KNP, and URP, non-fertilization with crop cultivation, and bare land without cropping. Moreover, the comparative yield analyses were also made among the treatments in Shunyi District, China.

## Materials and Methods

### Site Details

Shunyi District (40.1303°N, 116.6547°E) is situated in the Northeast of Beijing, having a total area of 1,021 km^2^, including 5.0 × 10^5^ ha agricultural lands under cereal crops, vegetable farms, fruit gardens, and grasslands for animal husbandry. The mean annual temperature is 11.5°C with hot and humid summer and with cold and dry winter. Mean annual precipitation is 600 mm but mostly occurs during monsoon (July–August) and thereby, irrigation is essential for crop production (Ṙuz̆ic̆ and Hansen, 1975; Geng et al., 2015).

**FIGURE 1:**
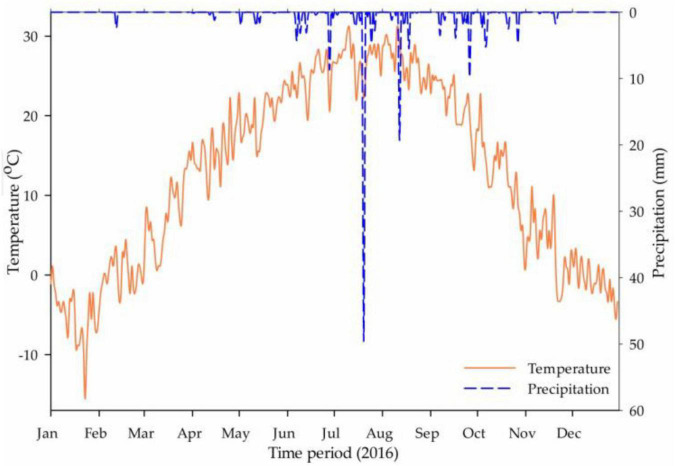
The average daily temperature (°C) and rainfall event (mm) recorded in the Shunyi District in the year 2016 (*source:* Weather Underground Online Record).

The rainfall events and daily temperature for the experiment year (2016) are presented in Figure 1 and soil texture details from 0 to 100 (0–20, 20–40, 40–60, 60–80, and 80–100) cm soil profile are given in Table 1. The experimental field had installed a drip irrigation system and scheduled on the demand basis but the same for all treatments.

### Experiment Procedure

Repeat Soil Core Method (RSCM) is used in this study to evaluate the ammonium, nitrate, pH, EC, and total PAN differences with respect to the cropping season and soil profile (0–100 cm). RSCM was used to evaluate the surface and subsoil physical and chemical properties as the number of soil profile samples was taken at different locations from the experimental field and the same procedure was repeated after a pre-defined time of 1 month. The number of samples taken in a single collection from treatment was considered as repetitions for a parameter. In this study, nine repetitions were taken for a single layer in a plot. The changes in the properties of the soil were analyzed with respect to time, treatment, and soil profile depth. Moreover, this method is easy to handle and broader regions can be covered in actual field conditions. Any fertilization in soil may significantly affect the plant available N within a soil profile of 0–100 cm (Rayment and Higginson, 1992) and provides realistic analysis if distributed in small sectional layers, such as 0–20, 20–40, and 40–60.

This experiment was conducted in the field, having five plots of equal size 25 m × 5.25 m and had three rows of 1.75 m spacing as the split plot design. Five treatments were applied in five plots, including bare soil with no fertilizer and no crop planted (NVP), Chinese cabbage plantation with either no fertilizer (CTP), urea at a rate of 300 kg N/ha (URP), potassium nitrate at a rate of 300 kg N/ha (KNP), or chicken manure at a rate of 300 kg N/ha (CMP). Soil samples were collected and divided into five equal sections 0–20, 20–40, 40–60, 60–80, and 80–100 cm along with the Gouge Auger (6 cm diameter and 100 cm length). Chinese cabbage (*B. rapa* subsp. *pekinensis*) was transplanted as 0.25 m plant to plant and 1.75 m row to row distance after the 1 week of fertilization. A single plot contained 300 plants sowed in three rows. NVP plot was left without any interference after secondary tillage. Soil samples were collected at the time of pre-planting and fertilization (t_0_), during cropping season as 1 month after fertilization (t_1_), 2 months after fertilization (t_2_), and 3 months after fertilization (t_3_) just after harvesting. At each time point, nine soil cores for 1 m soil depth in each plot was collected at three repetitions from each row and overall nine repetitions. Samples were marked and transferred in an ice-filled container from the field site to the laboratory. Nitrate and ammonium analyses were made using Lachat flow injection (QuikChem^®^ 8500 Series 2 FIA System, United States with 0.5% accuracy and reproducibility) (Butterly et al., 2013). For nitrate and ammonium analysis, the soil was extracted using 2 M KCl at a 5:1 ratio. For soil, pH, and EC, air-dried soil samples were passed through a 2 mm sieve, mixed with de-ionized water solution at a ratio of 5:1, and shaken for 1 h at 15 rpm (McLaughlin, 2009). A digital pH and EC meter (Mettler Toledo™ S230 SevenCompact™ with ±0.5% accuracy) were calibrated using a standard solution of 1,413 μS/cm at 25°C for EC and buffer solution was used to calibrate at pH 4, 6, and 7 Electrodes were washed with distilled water and wiped with tissue after each reading.

### Statistical Analysis

The data were adjusted by subtracting each value for the soil pH, EC, nitrate, ammonium, and PAN at t_1_, t_2_, and t_3_ from the baseline value collected at pre-plant (t_0_). Positive values represent an increase from the baseline (t_0_) and negative values represent a decrease relative to the baseline (t_0_). Data were analyzed by two-way analysis of variance (ANOVA) using Statistix v.8.1 software. Multiple comparisons were made using the Tukey HSD with 0.05 alpha (α = 0.05) for the time, treatment, and time-treatment interaction. The yield data analysis was also made by the one-way ANOVA. The graphical presentation was being made using GraphPad Prism v.6.01 and SigmaPlot v.13.

## Results

### Soil pH

**FIGURE 2:**
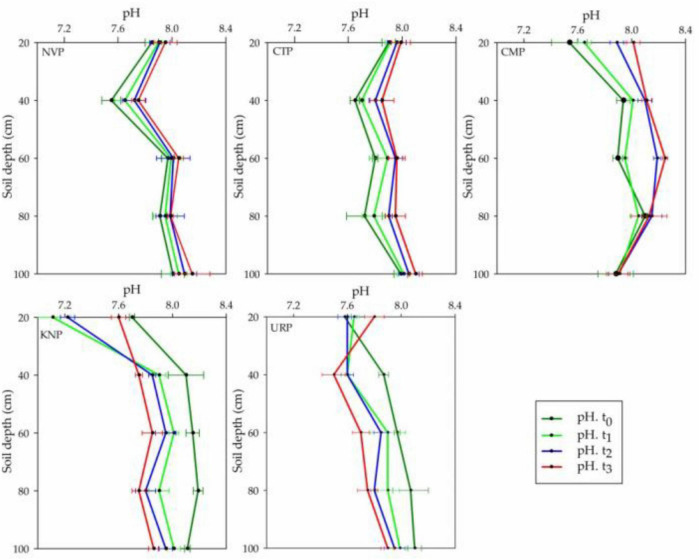
Mean values with standard error bar of soil pH in soil profile (0–100 cm) for the treatments NVP, CTP, CMP, KNP, and URP before (t_0_) and after fertilization at t_1_ (1 month), t_2_ (2 months), and t_3_ (3 months) in the experimental fields of Shunyi District, China.

In the study area of Shunyi District, soil pH ranges from 7.0 to 8.5 which is a suitable range for crop production. The pH variations were analyzed more in the above soil layers from 0 to 80 cm than in 80–100 cm for all treatments as shown in Figure 2. CTP, NVP, and CMP showed an increasing trend, while URP and KNP showed a decreasing trend relative to the baseline t_0_ values. Soil layer from 80 to 100 cm showed minimum variations in NVP, CTP, and CMP as compared with URP and KNP. In soil profile, the mean variations for all treatments were not significantly different at the time t_1_, t_2_, and t_3_ in the soil profile 0–100 cm, whereas treatments were significantly different among each other as given in Table 1.

**TABLE 1:** The results of two-way ANOVA (split plot design) was used with the Tukey HSD (α = 0.05).

Treatment	pH				EC			
	t_1_	t_2_	t_3_	Mean	t_1_	t_2_	t_3_	Mean
CTP	0.05b	0.12ab	0.16ab	0.14a	−2.8f	−1.4def	−1.86ef	−2.02c
NVP	0.05b	0.09ab	0.12ab	0.11a	−3.4f	−2.6f	−1.13cdef	−2.03c
CMP	0.04b	0.18ab	0.21a	0.09a	3.66bcdef	8.67abcde	9.33abcd	7.22b
KNP	−0.21cd	−0.28d	−0.29d	−0.27c	11.67ab	18.27a	18.87a	16.27a
URP	−0.11c	−0.16cd	−0.19cd	−0.15b	9.4abc	14.60a	9.2abcd	11.07b
Mean	−0.037a	−0.015a	0.02a		3.70b	6.88ab	7.50a	

*The lowercase letters (a, b, c, d, e, f) show the level of significance in treatments and times for the soil pH and EC at p < 0.05.*

### Soil Electrical Conductivity Variations

**FIGURE 3:**
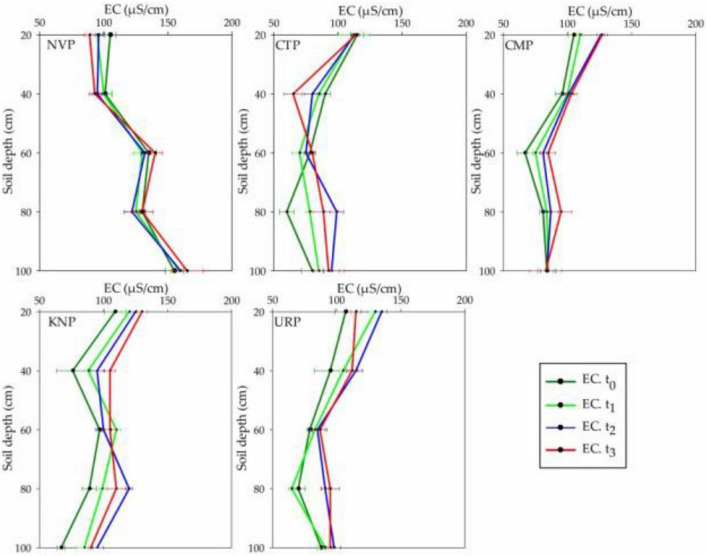
Soil electrical conductivity (EC) mean values with standard error bar in soil profile (0–100 cm) at t_0_, t_1_, t_2_, and t_3_ for the treatments during the Chinese cabbage production in Shunyi District, China.

The mean EC variations in soil profile from 0 to 60 cm showed a decreasing trend in NVP and CTP while increasing trend in CMP and KNP after the pre-planted t_0_ up to the harvest time t_3_. URP represented a different result from the other treatments as the mean EC increased up to t_2_ and thereafter decreased at t_3_. Maximum variations were recorded in the upper layers from 0 to 40 cm than 60 to 100 cm for all the treatments. In soil layers from 60 to 100 cm, uneven addition was found in all treatments from the baseline t_0_ as shown in Figure 3. Statistically, variations in the fertilized plots (CMP, KNP, and URP) were significantly different from non-fertilized plots (CTP and NVP) with maximum variations in KNP and minimum in CTP in the soil profile 0–100 cm as given in Table 1. In addition, the mean variations for all treatments were significantly different with respect to the time as maximum recorded at t_3_.

### Soil Nitrate Variations

**FIGURE 4:**
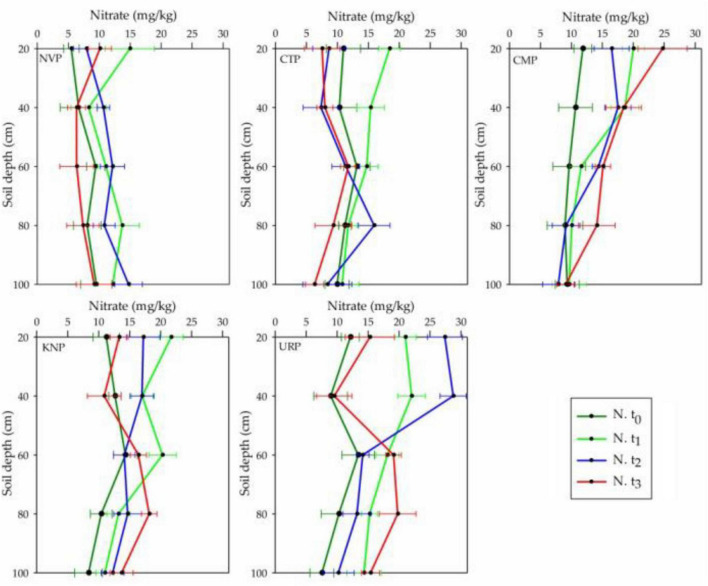
Nitrate mean and standard bar values in the different layers of soil profile 0–100 cm in the treatment plots of Chinese cabbage for the time periods t_0_, t_1_, t_2_, and t_3_ in the cropping season at Shunyi District, China.

Nitrate concentrations were recorded in the declined order from surface to bottom layers in the soil profile (0–100 cm) in all treatment plots at the baseline time t_0_, whereas at t_3_ (after harvest) CTP, NVP, and CMP represented the incline but URP and KNP decline trends as shown in Figure 4. It can be analyzed that nitrate level increased in layers 60–100 cm for all fertilized plots while decreased in non-fertilized plots. After harvesting, nitrate was recorded in CMP more than KNP and URP in the upper soil layer of 0–40 cm but not during the cropping season t_1_ and t_2_. The mean variations in the soil layers 0–40 cm were higher in URP as compared with KNP and CMP. In the soil profile 0–100 cm, the mean variations in nitrate were significantly different in all plots with respect to time and treatment as given in Table 2. The mean negative values of the CTP indicated that nitrate was decreased in the soil profile (0–100 cm) from the values recorded at the baseline t_0_, while the maximum increase was found in the URP. The mean values for all treatments recorded at t_2_ and t_3_ were less than t_1_ which presented the reduction in nitrate concentrations with the passing time.

**TABLE 2:** Presents the two-way ANOVA (split plot design) with the Tukey HSD (α = 0.05) for nitrate and ammonium ions in soil profile 0–100 cm.

Treatment	Nitrate				Ammonium			
	t_1_	t_2_	t_3_	Mean	t_1_	t_2_	t_3_	Mean
CTP	3.08bcde	−0.79ef	−2.53f	−0.08c	−1.49def	−1.49def	−2.92f	−2.31c
NVP	4.2abcd	3.42bcdef	0.04def	2.55b	−0.53cdef	−2.71ef	−3.13f	−2.13c
CMP	3.9abcd	3.00cde	6.20abc	4.36b	3.82abc	7.94a	3.92ab	5.22a
KNP	5.24abc	3.65abcde	3.09bcde	3.40b	4.46ab	1.50bcde	1.71bcd	2.56b
URP	7.68ab	8.26a	5.35abc	7.1a	3.54bc	5.15ab	3.33bc	4.01ab
Mean	4.82a	4.82a	2.43b		1.96a	1.87ab	0.58b	

*The lowercase letters (a, b, c, d, e, f) show the level of significance in treatments and times at p < 0.05.*

### Soil Ammonium Variations

**FIGURE 5:**
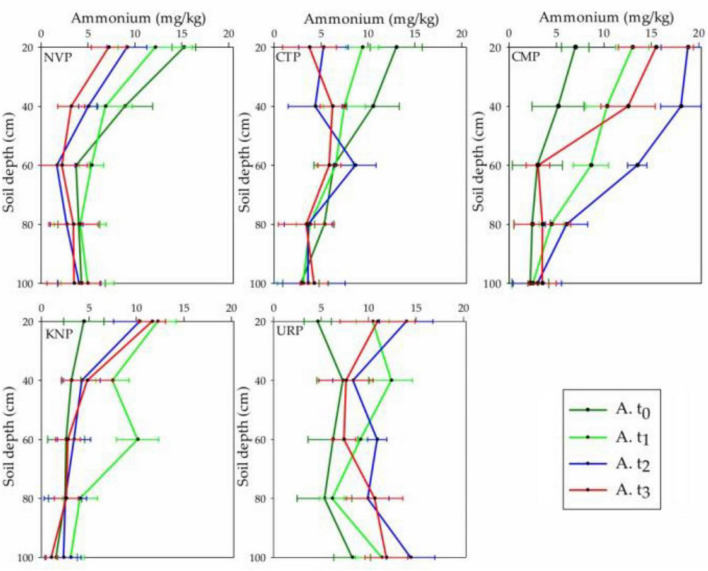
Ammonium levels in different soil layers (0–100 cm) for the treatments at the time periods t_0_, t_1_, t_2_, and t_3_ in the cropping season of Chinese cabbage production at Shunyi District, China.

Ammonium concentrations were increased from the baseline t_0_ in the fertilized plots (CMP, KNP, and URP) in the soil layers from 0 to 60 cm, while decreased in non-fertilized plots (NVP and CTP) at t_1_, t_2_, and t_3_. The mean values at t_3_ in CMP and URP were higher in the soil layers 60–100 cm from baseline t_0_ values which represented the accumulation of ammonium in these layers after the cropping season as shown in Figure 5. In CMP, the ammonium level increased significantly in t_1_, gained peak level at t_2_ and declined at t_3_, whereas URP and KNP gained peak levels in t_1_ and thereafter showed declining trends lasting up to the harvesting period (t_3_) in soil layer 0–40 cm. The maximum increase in mean values was observed in lower layers (40–100 cm) for CMP and URP as compared with KNP in t_1_. In soil layers up to 60 cm, CMP had shown maximum increase but the deeper layers of URP showed peak values. The negative mean values in non-fertilized plots (NVP and CTP) represented loss, whereas positive values in fertilized plots (CMP, KNP, and URP) represented the gain of ammonium in the soil layers as given in Table 2. Statistically, ammonium concentrations were significantly different with respect to the treatment and time in the soil profile 0–100 cm.

### Total Plant-Available Nitrogen

**FIGURE 6:**
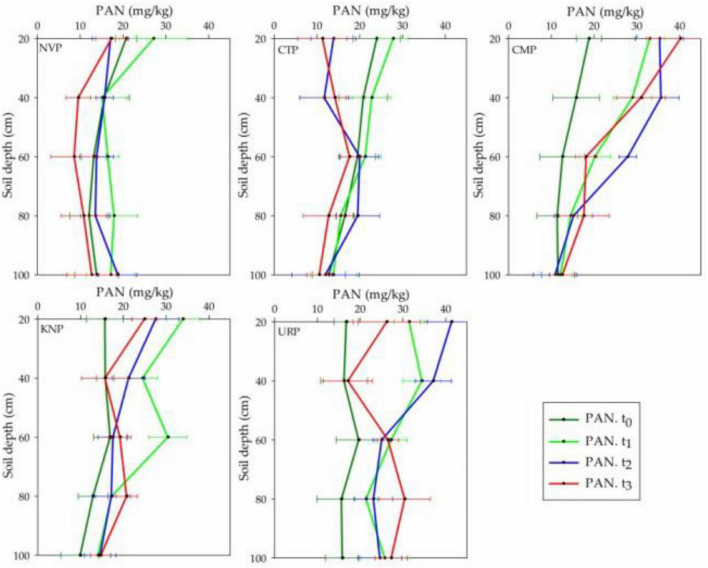
Plant-available nitrogen (PAN) (nitrate + ammonium) variations in soil profile (0–100 cm) for the treatments NVP, CTP, CMP, KNP, and URP before fertilization (t_0_) and after 1 month (t_1_), 2 months (t_2_), and 3 months (t_3_) of fertilization during the Chinese cabbage production in Shunyi District, China.

Plant-available nitrogen represented the declining trend in soil layer 0–20 cm from t_0_ to t_3_ in CTP, t_1_ to t_3_ in KNP and NVP, while inclined to the maximum level at t_2_ in CMP and URP as shown in Figure 6. Statistically, it was recorded that all treatments showed significant variations with respect to time, treatments, and their interaction as shown in Table 3. PAN was significantly increased in all fertilizer treatments and decreased for a non-fertilizer plot in the whole soil profile. After harvesting, peak PAN levels were observed in soil profiles 60–80 cm for all treatments. Urea presented the maximum for the soil layers 0–40 cm at t_1_ and for 20–80 cm at t_3_ than KNP and CMP. The negative mean values were recorded in only CTP which showed decreased PAN in the soil profile 0–100 cm from the baseline t_0_ values.

**TABLE 3:** Presents the two-way ANOVA (split plot design) with the Tukey HSD (α = 0.05) for the plant-available nitrogen (PAN) in soil profile 0–100 cm.

Treatment	PAN			
	t_1_	t_2_	t_3_	Mean
CTP	1.52cdef	−3.31ef	−5.46f	−2.42c
NVP	3.67bcde	0.714def	−3.3ef	0.37c
CMP	7.76abcd	10.97ab	9.89ab	9.54ab
KNP	9.80ab	5.34bcd	4.82bcd	6.67b
URP	11.23ab	13.42a	8.69abc	11.11a
Mean	6.79a	5.43a	2.93b	

*The lowercase letters (a, b, c, d, e, f) show the level of significance in treatments and times at p < 0.05.*

### Total Yields

**FIGURE 7:**
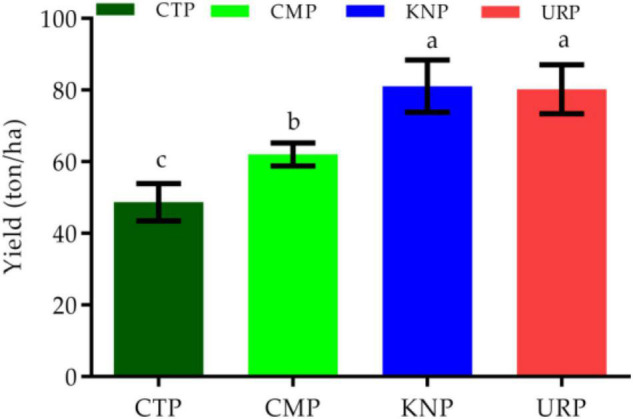
Chinese cabbage yield in different treatments with standard error bar and letters (a, b, and c) presenting the statistical significant variation (*p* < 0.05).

Every plot (except NVP) had 300 plants at the time of harvest. From every row, two average plants were taken as representative values for yield analysis. Thus, every plot had 6 values and these values were used to convert yield per hectare. Overall, the yield was significantly higher in all fertilizer plants than in control. KNP and URP (chemical fertilizers) presented significantly higher yields than CMP (organic fertilizer) as shown in Figure 7. Chinese cabbage yield followed the order from maximum to minimum as URP > KNP > CMP > CTP.

## Discussion

A cabbage root zone was spread over 100 cm of soil and rapidly decreased with the increasing soil depth that was able to effectively absorb the part of soil nutrients in the root zone. As a result, we selected soil depths from 0 to 40 cm in this study. The results showed that the soil nitrogen content increased with an increasing soil depth from 0 to 30 cm in 25 days after planting. In extremely well-drained soils, the content was mainly distributed in the topsoil (0-20 cm) 43 days after planting, and the soil nitrogen content was reached to 71.98 mg kg^–1^. The same result was obtained when the soil nitrogen concentration was reduced from 30 cm. Most parts of the soil nitrogen content remained in the topsoil (0–50 cm), which was associated with several root parameters (Yousaf et al., 2016). Soil pH temporal variation in a particular cropping system depends upon the plant species or crop grown (Curtin and Trolove, 2013), nitrogen fertilization source (Bird and Rapport, 1986), and the buffering capacity of the soil (Min et al., 2016). In many regions of the world, it was observed that NL after inorganic N fertilizers may cause considerable acidification in the soil such as in Atlantic Canada, 74% acidic effects came from N fertilizers and 26% from acidic rain (Kemmitt et al., 2008). An increase in the nitrate ions may decrease pH in a soil profile exponentially. Hence, nitrate accumulation in the soil profile is a substantial factor affecting soil acidification (Duruigbo et al., 2007). Soil pH is the main regulating factor of soil microbial activities and the rate of soil carbon and N cycling that may be disturbed by urea acidification (Eigenberg et al., 2002). In this study, the same results were found in the urea plot as pH declined in the first month and thereafter, returned to the initial values. In previous results, the poultry manure has the ability to increase the pH level of soil when it was treated as 15 ton/ha (300 kg N/ha with 2% N) (Ruter, 1992).

Statistically, overall temporal EC variations in the soil profile before, during, and after plantation and fertilization were observed significantly in all plots but the maximum increase was observed in KNP. Potassium nitrate is considered to be the salt fertilizer and has the ability to change the soil EC (Djurhuus and Olsen, 1997). Thus, significant variation in soil EC might be occurred due to the potassium nitrate fertilization. Organic and inorganic fertilization in agricultural soil raises its EC (Sims et al., 1995; Sainju et al., 2019) which might be the reason for high EC in KNP, URP, and CMP as compared to CTP and NVP.

The nitrate levels in the first 2 months were increasing in NVP and thereafter declined to initial values in the soil profile which might be due to the increasing mineralization of bare tilled soil. The same results were noted by Sainju et al. (2019) in Piarco Series Soil (West Indies) while analyzing the temporal differences between nitrate concentrations in the soil profile from 0 to 90 cm in the early and late rainfall seasons. Nitrate in CTP increased in the first month but declined up to the harvest period, such as the lowest concentrations were measured in all layers of the soil profile at the end of the cropping season. It might be happened due to the plant growth which might increase the nitrate uptake from the root zone as well as decreased the total N budget and mineralization. It was recorded that nitrate concentration was increased in all the layers of soil profile as compared with the initial values in CMP, particularly after 2 months of chicken manure application. It was observed that chicken manure contained a high rate of available N and a larger fraction in organic form which needed further mineralization to release ammonium or/and nitrate ions. The time rate of poultry manure mineralization was not fixed, such as Azeez and Van Averbeke (2010) found that 48% of the organic N in poultry manure was released within 10 weeks during the laboratory experiment, whereas Bitzer and Sims (1988) reported 40% during 18 weeks. Fan et al. (2003) reported maximum nitrate available in the soil about 10–15 weeks after broiler manure application (Rong and Xuefeng, 2011). In this study, higher nitrate concentration after cropping season might be happened due to the slow mineralization rate. In KNP soil layers from 0 to 60 cm showed that the nitrate level increased to a certain limit in the first 2 months and thereafter decreased up to nearly initial concentration whereas, in soil layers 60–100 cm, the nitrate level increased up to the end of the cropping season. Few researchers reported that the balance amount of potassium in the soil might withhold nitrate ions, increase nitrate uptake in the plant body, and decrease NL (Gabriel et al., 2012; Zhou et al., 2016). Sainju et al. (2019) worked out the adsorption effects of potassium ions with nitrate ions and found minimum adsorption in the soil surface layers from 0 to 15 cm as compared with subsoil layers. Furthermore, subsoil layers from 0 to 90 cm showed maximum adsorption effects as withholding the maximum amount of nitrate in the field experiment. Therefore, it might be the reason why subsoil layers from 60 to 100 cm presented nitrate in raising trend up to the harvesting time. In soil profile from 0 to 40 cm in URP treatment, the nitrate level increased after the first 2 months and turned back to the initial level thereafter, in the soil layers from 40 to 60 cm inclined up to the harvesting period as shown in Figure 5. Rong and Xuefeng (2011) affirmed in their experiment that urea fertilization had a positive relation with nitrate accumulation in soil profiles from 0 to 100 cm. In this study, nitrate incremented in soil layers of 0–40 cm and lasting up to 2 months, was previously found, such as Sainju et al. (2019) reported non-significant variation in soil profile within 4 weeks subsequent to urea fertilization but thereafter, found a significant increase in the nitrate level.

It was obvious from the previous studies that the maximum mineralization of organic matters and the nitrification of residual nitrogen occurred in the fallow period or bare soil without vegetation after harvesting (Bishop and Manning, 2010), which might be the cause of ammonium level raised in CTP and NVP at t_1_ in the soil profile. In Shunyi District, clay level was found in increasing order with the soil depth as presented in Table 4, which might be constructed the plow pan in the subsoil and raised the ammonium level in lower layers. The temporal variations in ammonium levels before and after cropping season in CTP showed that the final values were three times lesser than the initial values in the 0–60 cm soil profile which might be happened due to lower soil mineralization rate as compared with ammonium utilization (nitrate conversion, volatilization, and plant uptake). In KNP, it was recorded that the ammonium level was decreased in 0–60 cm soil layers before and after cropping season. Meanwhile, a little increment was noted in 60–100 cm soil layers that might be due to the potassium nitrate which promotes the nitrate concentration in soil, not ammonium. Thus, the overall ammonium in soil decreased during the cropping season due to the plant consumption and utilization as nitrate conversion and volatilization. Soil layers 0–60 cm in CMP made available higher ammonium concentration throughout the cropping season as compared with initial values (before fertilization) whereas, soil layers from 60 to 100 cm showed negligible variation. Similar results were observed in the study of Perego et al. (2012). Temporal increment in ammonium level in URP treatment was disappeared in soil profile from 0 to 60 cm but lasted in 60–100 cm after harvesting. As urea addition might be the cause of significantly increased ammonium (Xiong et al., 2012) up to the maximum limit within 4 weeks after fertilization in the presence of moisture, whereas subsequent rainfall or irrigation might drag down the urea concentration in the soil profile (Gai et al., 2016). In this study, it was observed that the total PAN in the soil profile (0–100 cm) increased to maximum level after 1 month (September) of fertilization, not only in fertilized plot but also in without fertilized (NVP and CTP) plots which might be due to the heavy rainfall occurred during this month as shown in Figure 1. It was proved that heavy rainfall events increased nitrate and ammonium in the soil profile (0–100 cm) (Zhang et al., 2013).

**TABLE 4:** Soil textural classes in the soil profile 0–100 cm (according to IEDA laboratory results).

Depth (cm)	Sand (2–0.05 mm) %	Silt (0.05–0.002 mm) %	Clay (<0.002 mm) %	Soil type
0—0	48.40	45.38	6.22	Loam
20–40	47.01	39.10	13.89	Loam
40–60	45.75	36.43	17.82	Loam
60–80	44.13	35.71	20.16	Loam
80–100	41.52	39.82	18.66	Loam

It was observed that the maximum Chinese cabbage yield was produced in fertilized plots than control plots but significant variations were also observed among the fertilizer treated plots as well. Maximum yield was observed in URP than KNP but both have significantly higher values as compared to CMP. It might be possible that because poultry manure had the slow N release at the time of high plant requirement as previously reported in a similar study (Li et al., 2021).

## Conclusion

Chinese cabbage produced maximum yields in fertilizer treated plot because of optimum PAN level during the cropping season. Chinese cabbage had shallow root depth, required frequent irrigation, and optimum PAN in the upper soil layer 0–40 cm for optimum production. Therefore, total PAN concentration and PAN lasting time in a root zone (0–30 cm) are important while selecting fertilizer type and crop type. In our experiment, the variations in soil pH, EC, nitrate, ammonia, and PAN in soil profile 0–60 cm with respect to initial values for all treatments were larger as compared to variations in soil profile 60–100 cm. PAN level as ammonium was found maximum in CMP as compared to the other treatments. CMP showed a higher ammonium concentration in the upper soil layer during the cropping season and increased the potential use of PAN and decreased the potential NL as compared to KNP and URP. Bare land without plantation also caused to increase in PAN concentration in lower soil profile 0–60 cm. Higher PAN levels as compared to initial values in soil profile 60–100 cm showed the N losses occurred after all treatments and had the potential to contaminate the groundwater resources except for the CTP. Furthermore, Chinese cabbage productions without fertilization significantly dragged down the PAN level in soil layers 0–60 cm as well as reduced crop yield. For a single cropping season, pH variations in the soil profile of 0–100 cm were significant during and after cropping season with respect to time and treatments, although these were very minute as compared to the initial values. Similar results were observed for EC as well. Overall, the soil EC and pH values were in the decreasing trend for KNP and URP, whereas in increasing trend in NVP, CMP, and CTP. Therefore, a single cropping season may vary notable differences in nitrate, ammonium, and PAN levels in the soil (Shunyi District, Beijing, China) profile with smaller differences in soil pH and EC.

## Data Availability Statement

The raw data supporting the conclusions of this article will be made available by the authors, without undue reservation.

## Author Contributions

RA designed the experiment methodology and wrote the manuscript. YL was the supervisor and project administrator, assisted to correct the methodology, and validate the design. JD, WL, and SA supported data curation and laboratory work. LM helped in reviewing and editing. RA made the software analysis with the assistance of LM, SA, and WL. WA and AA have done the data analysis and curated and helped in manuscript writing. All authors contributed to the article and approved the submitted version.

## Conflict of Interest

The authors declare that the research was conducted in the absence of any commercial or financial relationships that could be construed as a potential conflict of interest.

## Publisher’s Note

All claims expressed in this article are solely those of the authors and do not necessarily represent those of their affiliated organizations, or those of the publisher, the editors and the reviewers. Any product that may be evaluated in this article, or claim that may be made by its manufacturer, is not guaranteed or endorsed by the publisher.
